# Syntactic mixing across generations in an environment of community-wide bilingualism

**DOI:** 10.3389/fpsyg.2015.00082

**Published:** 2015-02-17

**Authors:** Sabine Stoll, Taras Zakharko, Steven Moran, Robert Schikowski, Balthasar Bickel

**Affiliations:** Department of Comparative Linguistics, University of ZürichZürich, Switzerland

**Keywords:** code-switching, bilingualism, language mixing, language acquisition, language contact, Chintang, Nepali

## Abstract

A quantitative analysis of a trans-generational, conversational corpus of Chintang (Tibeto-Burman) speakers with community-wide bilingualism in Nepali (Indo-European) reveals that children show more code-switching into Nepali than older speakers. This confirms earlier proposals in the literature that code-switching in bilingual children decreases when they gain proficiency in their dominant language, especially in vocabulary. Contradicting expectations from other studies, our corpus data also reveal that for adults, multi-word insertions of Nepali into Chintang are just as likely to undergo full syntactic integration as single-word insertions. Speakers of younger generations show less syntactic integration. We propose that this reflects a change between generations, from strongly asymmetrical, Chintang-dominated bilingualism in older generations to more balanced bilingualism where Chintang and Nepali operate as clearly separate systems in younger generations. This change is likely to have been triggered by the increase of Nepali presence over the past few decades.

## 1. Introduction

One of the key effects of sustained language contact is code-switching, i.e., the switching from one language to another either within or across utterances. Over time this can lead to language change, sometimes resulting in a mixed code with new structures (Muysken, 2007). Here, we explore changes in code-switching during language acquisition and more broadly across all generations in a population with community-wide bilingualism. Even though there are no exact numbers, this kind of bilingualism is probably the most common one in the world, partly as the result of national languages and *lingua franca* encroaching on vernaculars worldwide. Despite this, most research on acquisition and trans-generational patterns in bilingual situations has so far focused not on community-wide but on individual bilingualism, where a single child, or a small group of children, is raised bilingually in an otherwise monolingual community.

Research on community-wide bilingualism opens important windows on key processes of acquisition and the interplay between acquisition and language change. Regarding acquisition, research on this kind of bilingualism allows fresh insights into the still unresolved question of whether bilingual acquisition is characterized mostly by straightforward input mirroring or mostly by a gradual increase of proficiency in the dominant language with a concomitant decrease of on-the-fly borrowing (Genesee, 2001). If bilingualism is community-wide and stable, one might expect more cases of straightforward mirroring, with the result that children can be expected to show similar amounts and types of code-switching as adults [as found for example in case-studies by Paradis et al. (2000) and Allen et al. (2002)]. But there are likely to be many confounding factors, such as the effective balance between languages in use, that might create different scenarios, and a proficiency-based trajectory of language learning remains a possibility.

Another window opened by community-wide bilingualism concerns the interplay between language acquisition and language change, i.e., developments across generations. Contact with other languages and bilingualism are well-established factors in such developments and have rightly been of great concern in historical linguistics. But the kind of bilingualism that is typically relevant for this is not individual but group-wide or even community-wide bilingualism. Here, many questions remain unresolved, such as: Is children's bilingualism relevant for change, or only adolescent and adult bilingualism? How does bilingualism relate to language change when the nature—e.g., the balance—of bilingualism changes itself across generations? What are the social factors favoring effects of bilingualism on language change? For these and other questions we mostly lack sufficiently detailed research, although new studies in this area keep offering challenging findings. A case in point are recent discoveries that in community-wide bilingual situations, children play an unexpectedly strong role in language change: qualitative analyses of Light Warlpiri (O'Shannessy, 2005, 2012) and Gurindji Kriol (McConvell and Meakins, 2005), for example, show that children can have a significant impact in language change in these situations, even resulting in the emergence of new mixed languages.

A deeper understanding of bilingual acquisition and trans-generational developments poses three challenges. First, it is essential to broaden the scope to other situations of community-wide bilingualism because there is extreme variability in the type and amount of code-switching across such communities (e.g., Poplack, 1980, 1987; Muysken, 2007; Matras, 2009). Second, it is critical to move beyond qualitative analyses and to study patterns of code-switching in naturalistic recordings over time (Poplack, in press), systematically comparing children's productions with the surrounding speech they are exposed to, and comparing generations with each other. Third, since any possible effect of code-switching on language change depends on constraints imposed on code-switching, it is essential to review proposed universal constraints (e.g., Poplack, 1980, in press; Sankoff and Poplack, 1981; Di Sciullo et al., 1986) in the light of new and typologically more varied datasets.

In this paper, we respond to these three desiderata by examining code-switching in a hitherto unstudied area, involving contact between two languages that have very different structural profiles than the ones studied so far and that also strongly diverge from each other: Chintang (ISO 639-3 ctn), a polysynthetic Tibeto-Burman language, and Nepali (nep), an Indo-European language that strongly diverges in structure from its better-known relatives in Europe. Second, we move to quantitative analyses and explore patterns in a large corpus of natural conversational data with respect to differences in code-mixing behavior over time in acquisition by children and across generations. Third, we test proposed constraints against syntactic integration of single-word vs. multi-word insertions. Here, findings from previous research suggest that multi-word insertions resist syntactic integration (Poplack, 1987, see papers in Poplack and Meechan, 1998b), unless insertions become so frequent that in fact a new mixed language has emerged. We exclude from our purview detailed research on phonological integration and on the socio-linguistic functions of code-switching. These areas are left for future research.

## 2. Contact situation and types of code-switching

Chintang is spoken in a small rural area in Eastern Nepal (centered at around 26°57′ N and 87°12′ E). Bilingualism is mostly asymmetric, i.e., Chintang speakers know Nepali, and there are only very few Nepali speakers who know Chintang. The indigenous language of the area is Chintang. Nepali came in only in the aftermath of a conquest by a Nepali-speaking kingdom in the late 18th century (Pradhan, 1991). A critical feature of this conquest was that military leaders compensated soldiers and allies for their services generally not by money or goods, but by granting land rights. This triggered relatively quick and intensive growth of Nepali presence and suggests that the current bilingualism has its roots about 5–7 generations ago. However, it is unclear how intense bilingualism was since earlier regimes in Nepal imposed strict rules of social segregation. Language contact has strongly increased over the past few decades, after the introduction of a school system in Nepali and the ever-growing presence of modern communication and broadcasting means. A critical change happened in the 1960s when the proportion of children in schools increased by about 60% in rural areas (Central Bureau of Statistics, 1977).

There are approximately 5000–6000 Chintang speakers and all of them are bilingual in Nepali. According to the UNESCO classification scheme (UNESCO Ad Hoc Expert Group on Endangered Languages, 2003) Chintang is endangered although not moribund: most children still learn the language and Chintang is still used in a large variety of daily contexts, especially at home. At the same time children encounter Nepali in many contexts from early on, since a number of native speakers of Nepali or speakers of other languages who use Nepali in daily interaction live in the area as well. Nepali is considered to be important for economic success, and it is also the only language of instruction in schools.

Chintang and Nepali are typologically very different in many dimensions. Chintang features complex morphology with clear-cut parts of speech. While nominals express case, number, and possession, verbs are characterized by a strong degree of synthesis, including agreement with one or two arguments and expressing a large number of categories including tense, aspect, mood, polarity, and many more or less paradigmaticized notions of event structure. This combines with heavily distributed (discontinuous) exponence and an intricate system of variable positioning of some affixes (Bickel et al., 2007), yielding over 1800 forms attested in our corpus (Stoll et al., 2012). Case and agreement frames are mostly based on ergative alignment, but a number of differential marking patterns and valency alternations such as unmarked antipassive- and passive-like constructions bring with them a high degree of syntactic flexibility (Schikowski et al., 2015). Syntactic phrases are basically head-final, and this is also true of clauses, but there, constituent order is relatively flexible. Complex sentences make ample use of non-finite forms including converbs and an infinitive involved in various kinds of raising and (backward) control constructions. Sentences are often nominalized for the purpose of clause combining or information structure manipulation.

Nepali shows considerably less morphology. Nouns carry case and number markers, verbs show subject agreement fused with tense/aspect and polarity. The coexistence of differential subject and differential object marking result in a variety of case frames that are furthermore split by conditions of tense, aspect, and finiteness (Schikowski, 2013). While Nepali also features a number of valency-changing alternations such as a passive, these alternations are fully marked. An important feature of Nepali are participial constructions that play key roles in clause combining and in the formation of a substantial set of compound and periphrastic tenses. One of the few areas where Nepali is similar in type to Chintang is linear order in the syntax, an observation we come back to in the Discussion Section.

We observe both single-word and multi-word code-switching to Nepali. In analyzing single-word insertions, we follow (Poplack, 1987, in press) and do not distinguish between a borrowing, i.e., a loan-word, and so-called nonce-borrowings or on-the-spot-borrowings. The classical distinction between loan-words and on-the-spot borrowings is based on the extent to which a word has been used in the community before it was recorded, but given the Zipfian expectations on word frequency, this leads to severe sampling problems, and so the extent of the adaption of words is very difficult to quantify. As a consequence, we simply collect all Nepali insertions in the speech of Chintang native speakers and then examine their syntactic integration. What we do distinguish is single-word vs. multi-word insertions. This is illustrated by the following data, where (1-a) shows single-word insertions and (1-b) a multi-word insertion. Along with morpheme-by-morpheme glosses we also include a line specifying the source language of each morpheme (C for Chintang, N for Nepali). The numbers in brackets indicate the age of the speaker and the reference ID of the example in our corpus^1^.

(1)     a.   *Abo ho-khi
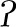
nɨŋ       pheri*
              now what-METHOD  again
              N     C-C                    N
              *khems-o-ko                            ni    naŋ?*
              hear-3[s]O-IND.NPST[.3sA] ASS but
              C-C-C                                   N     C
              “Now how does she hear that again?”
                                                          [64; CLDLCh3R01S03.074]
         b.   *Sak-ne-le                              tera   din*
               be.able-NPST.PTCP-ERG    13     day
               N-N-N                                   N      N
               *mai-hatt-a-k-e*.
               [3>]1nsi-wait.for-PST-IPFV-IND.PST
               C-C-C-C-C
               “The ones who were able to do so waited for us for
               13 days.”                                         [69; LH_Lal.0578]

All insertions generally show some amount of transphonologization, but we also note that the Nepali of Chintang speakers shows strong effects of Chintang phonology, not only when inserting Nepali into Chintang but also when speaking Nepali on its own. Regarding morphosyntactic integration, there are many cases where Nepali words are inserted as such, without any affixation. However, nouns and verbs can also be marked as insertions by a set of dedicated markers glossed as nativizer (NTVZ):

(2)        *Pahiro    rok-e              num-ma   jamma boll-a*
            landslide stop-V.NTVZ do-INF    all         effort-NTVZ
            N            N-C               C-C         N          N-C
            *numd-i-ne*.
            do-1pi[S]-OPT
            C-C-C
            “We should all make an effort to stop the landslide.”
                                             [adult, age unknown; ctn_prob_talk.0719]

In this example the NTVZ *-e* is attached to the Nepali verb stem *rok*- “stop,” while *-a* is found on the Nepali noun stem *boll*- “effort.” Verb NTVZs are obligatory on verb stems but they are not added to Nepali words with Nepali inflections (e.g., not to an imperative like *āijā*!^2^ “come!”). Noun NTVZs are optional and occur less often than verb NTVZs in the speech of younger or educated speakers. Insertion without any Chintang affix (of any kind, including NTVZs) is the majority case for nouns (72%, which also includes zero-marked nominatives) and is found with about half of verb stems (51%, which also includes zero-marked non-honorific imperatives like *āijā*!) across the entire corpus.

We also encountered complex combinations with Nepali modals or auxiliaries stacked on Chintang clauses:

(3)      a.   *Kok thuk-ma   por-ch-a          ni     maha
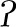
?*
                rice cook-INF must-NPST-3s ASS QTAG
                C    C-C         N-N-N              N     C
               “We really should cook rice, shouldn't we?”
                                                               [26; ctn_Fut_pln.354]
          b.    *Huŋ-go      ma
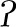
mi-ce=le
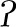
*
                 MED-NMLZ person-ns=only
                 C-C              C-C=C
                 *u-kos-a-yakt-e                              thi-yo*.
                 3pS-wander-PST-IPFV-IND.PST be\PST-PST.3s
                 C-C-C-C-C                                  N-N
                 “Only those people were wandering around.”
                                                                     [40; pear_9-3.052]

In (3-a), the modal verb *porcha* (transphonologized from Nepali *parcha*) governs a Chintang infinitival clause. The construction corresponds to a frequent pattern in Nepali where *parcha* “must, should” governs infinitival clauses, as well, i.e., (3-a) has a literal word-by-word translation in Nepali (*bhāt pakāunu parcha*). In (3-b), the Nepali auxiliary *thiyo* modifies a fully inflected past-tense verb in Chintang. The use of the auxiliary for past tense follows a regular pattern in Nepali, but there it would follow a participial rather than a finite form. (Singular number agreement here follows a common pattern in Eastern dialects of Nepali; see Genetti, 1999).

These data suggest strong syntactic integration since Nepali items govern or modify Chintang elements. This syntactic integration can show concomitant morphological exponence, as exemplified by the following data:

(4)     a.   *Ba       akka cah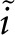     euta       sahayok-be-ko*
               PROX 1s     RETRV one.CLF help-LOC-NMLZ
               C         C      N          N            N-C-C
               *rup-be       khatt-u-ŋs-u-h-e*.
               form-LOC take-3[s]O-PRF-3[s]O-1sA-IND.PST
               N-C           C-C-C-C-C-C
               “I have taken this as one kind of help.”
                                                                  [35; Durga_job.0118]
        b.     *Hu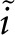-sa-ko                    lagi na*
                MED-OBL-LOC.NMLZ for  CONTR.TOP
                C-C-C                            N   C
                *ba-i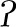          na                  naŋ jun=ai*
                PROX-LOC CONTR.TOP but    which=FOC
                C-C              C                    C      N-N
                *sastha-ŋa=yaŋ             bhon-uŋ      cainejo   ramro*
                organization-ERG=also say-OPT.1s RETRV good
                N-C=C                         N-C             N          N
                *man-de-na                ni     naŋ*.
                obey-NPST.NEG-3s ASS but
                N-N-N                      N      C
                “But as for this, in this place, I'd say no organization
                would really join in, don't you think.”
                                                              [72; ctn_prob_talk.0544]

In (4-a), the Nepali noun phrase [*euta sahayok*] “one (kind of) help” received a Chintang phrasal affix signaling locative case. The resulting locative phrase is in turn embedded as a modifier of the Nepali noun *rup*. Embedding is achieved with the help of the nominalizer *-go* (allomorph *-ko*), which is a regular means in Chintang of turning any kind of constituent into a modifier subconstituent of a noun phrase.

Example (4-b) shows an extended Nepali constituent marked by ergative case and an additive focus clitic [*junai sastha*]*-ŋa=yaŋ* “whichever organization.” While the ergative and the intervening expression [*bhonuŋ*] “I'd say” would lead one to expect continuation in Chintang, the speaker continues the sentence by switching into Nepali until the very last particle, which is again in Chintang.

In our analysis, we only focus on such morphologically marked cases of syntactic integration because our corpus is not yet parsed syntactically. This means that our report on morphologically marked syntactic integration below will inevitably underestimate the true amount of integration. Also, the unavailability of syntactic parses means that we cannot distinguish between multi-word insertions that form regular compositional phrase structure as opposed to insertions involving fixed expressions or idioms. Our impression is however that the insertions we found are not dominated by fixed expressions.

## 3. Materials and methods

### 3.1. Data

The data of this study comes from an audiovisual corpus consisting of adult conversations and a longitudinal corpus of language acquisition (Bickel et al., 2014)^3^. The entire sub-corpus used here contains 246,248 records, a unit that roughly corresponds to utterances as defined by phonological and syntactic integrity. The total number of words (as defined grammatically, not phonologically) is 612,672. There are 143 speakers ranging from a 9 month old child to a 79 year old woman (51 children up to and including age 12, 92 older speakers). All speakers and all interlocutors in the sub-corpus have Chintang as their dominant language.

**Figure 1:**
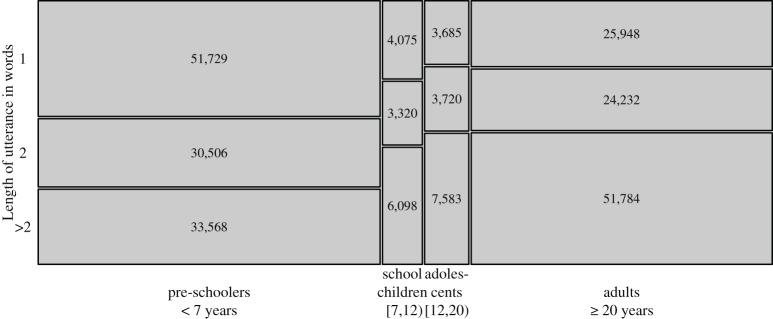
**Overview of data in the corpus, separated by age groups and utterance lengths**. Tile sizes are proportional to the sample sizes given inside them.

Figure 1 gives an overview of the number of utterances per age group, divided by utterance length. We have relatively fewer data on school children and adolescents (from age 7 up to age 20) because speakers of this age group were not usually present at the homes where the recordings took place.

The adult corpus comprises conversations about everyday topics of daily life. Speakers participate randomly in multiple sessions and were generally given no further instructions, except that sometimes they were encouraged to talk. The acquisition corpus includes recordings of 6 target children learning Chintang and Nepali in their natural environment over a period of 18 months. Two children were 6 months, two children 2 years and two children 3 years old at the beginning of the study. For each child, recordings summed up to approximately 4 h per month, recorded within a specific week, distributed over several recording sessions.

The climate in Chintang is warm and thus recordings were made mainly outdoors, either on the veranda of the houses or in the gardens and fields, where children spend most of their time. Children in Chintang usually play or roam around with other children. Starting from around 8 months of age they are carried around by other children and are part of larger groups of children (see Lieven and Stoll, 2013 for children's socio-communicative environment in Chintang).

For the recordings, no instructions were given to the families other than that we were interested in the daily activities of children and their language development. Further, no restrictions were made concerning conversational partners or people present during recordings. As a result, we captured free play among a large number of children and natural conversational exchanges of many different people of varying ages.

Each recording was conducted by a Nepalese research assistant familiar with the community, together with a native speaker assistant of Chintang who was part of the community and familiar with the children recorded. The assistants were instructed to intervene as little as possible in the activities recorded. The recordings were done with a video camera equipped with a fish-eye lens fixed on a tripod so as to interfere as little as possible by moving the camera. An external microphone was used to improve recording quality. The recorded interactions of adults within this corpus were mostly conversations on various household topics, chatting, gossip, politics, etc.

### 3.2. Coding and statistical analysis

All utterances were transcribed, translated into Nepali and English, morphologically glossed and tagged for part-of-speech categories. For all speakers, we collected detailed metadata to the extent possible and linked these to the corpus. In addition, each morpheme in the corpus was coded for whether it comes from Chintang or Nepali, in the way illustrated by the inter-linear glossing in (1)–(4) above.

The data were analyzed by counting the number of Nepali lexical stems within utterances (records). For modeling, the counts were turned into proportions based on the total number of lexical stems in each record; for one-word utterances, insertions were coded as a binary choice. In order to estimate syntactic integration of single vs. multi-word insertions, we also coded each inserted string for whether or not it hosts any Chintang affix, subsuming under this definition both stem affixes and phrasal affixes, but excluding grammatically free but phonologically bound particles [see (3) and (4) for examples]. For single-word insertions, this coding is limited to utterances with at least two words and for multi-word insertions to utterances with at least three words since the respective insertions are not defined for shorter utterances. Affixation by “nativizers” (examples in 3) was counted in the same way as other affixation because all these patterns are side-effects of syntactic integration: Nepali items are treated as if they were native vocabulary, i.e., the speaker is not just switching into another language on the fly.

Insertions per utterance and Chintang affixation per insertion were submitted to generalized linear mixed-effect modeling (GLMM), assuming a binominal distribution with a logit link function for the choice between Nepali and Chintang in one-word utterances and for the presence of Chintang affixes in insertions, and a normal distribution with an identity link function for the proportion of insertions in longer utterances. All models were estimates using the package lme4 (Bates et al., 2014) in R (R Development Core Team, 2014). To assess the significance of predictors, we used likelihood ratio tests comparing successively simpler models, each fitted with maximum likelihood. Apart from the several fixed effects predictors that we report on below, all models contained random intercepts for speaker identity and recording session.

For detecting major breaks in development or between generations, we performed breakpoint (spline) regression within the GLMMs. In each case, we ran through all possible breakpoints and chose the breakpoint that minimizes the deviance of the model and then tested the evidence of the breakpoint by comparing a GLMM with vs. one without a breakpoint, again using a likelihood ratio test. In addition to this, we also made use of tools for Pearson residual analyses in the package vcd (Meyer et al., 2006). Finally, for detecting overly influential cases and potential outliers we made use of the tools provided by Nieuwenhuis et al. (2012).

## 4. Results

Overall, the proportion of Nepali insertions per Chintang utterance averages at 32%. However, the probability of insertions necessarily depends on the length of utterances, reflecting the binomial probability mass function: for example, a two-word utterance has a 0.5 chance of being half-Nepali, while a four-word utterance has 0.375 chance of being half-Nepali; or a one-word utterance has a 0.5 chance of being completely Nepali, while a three-word utterance only has a 0.125 chance of being completely Nepali. Given the heavy skew toward short utterances among the youngest speakers (Figure 1), it is important to distinguish specifically between kinds of shorter utterances: one-word utterances show a mean of 40% insertions, two-word utterances a mean of 29% and longer utterances a mean of 27%. There is virtually no difference between three-word and longer utterances, with 26.7% and 26.5%, respectively, and in the following we collapse them.

### 4.1. Age

**Figure 2:**
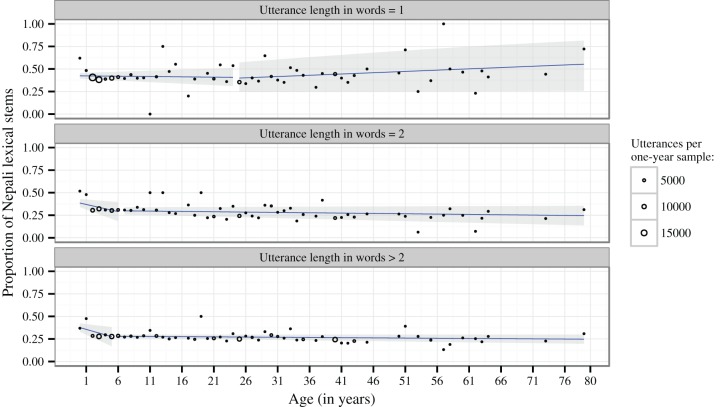
**Mean proportions of Nepali insertions per utterance over 1-year age intervals, controlling for utterance length**. The size of dots is proportional to the number of utterances within these intervals. The regression lines (blue) and 95% Wald confidence intervals (gray) represent the fixed effects estimates from generalized linear mixed models applied to invididual utterances and with recording session and speaker as random factors, assuming a binomial distribution for one-word utterances and a normal distribution elsewhere. Breakpoints are those that minimize the deviance of the models.

Mixed-effect modeling with utterance length (in number of words) and age (in years) as fixed factors reveals a significant interaction between length and age (χ^2^ = 20.25, *P* < 0.001)^4^. Visual inspection of interaction plots suggests that the interaction is best resolved by subsetting the data into the same distinction of one-word vs. two-word vs. multi-word utterances that is also the most relevant for the overall distribution of the data over age (cf. above). Figure 2 plots the proportions of Nepali insertions per utterance over 1-year age intervals, controlling for these three classes of utterance lengths.

Breakpoint analyses revealed significant breaks in linear trends at age 25 (one-word utterances, χ^2^ = 5531.2, *P* < 0.001), 6;9 (two-word utterances, χ^2^ = 9.72, *P* = 0.002) and 5;6 (longer utterances, χ^2^ = 12.67, *P* < 0.001). In two-word and longer utterances, the proportion of Nepali insertions significantly decreases over age below these breakpoints (two-word utterances: χ^2^ = 6.87, *P* = 0.009; longer utterances: χ^2^ = 7.50, *P* = 0.006), while there is no significant change above these breakpoints (both *P* > 0.05). The fixed effect estimates of these models are shown in Figure 2 by regression lines and 95% Wald confidence intervals.

One-word utterances show a weak but significant *increase* of being Nepali for speakers above age 25 (χ^2^ = 4.66, *P* = 0.031), but not below (χ^2^ = 0.22, *P* = 0.64). However, inspection of Cook's distances reveals that the effect for older speakers is overly influenced by only 6 (out of 67) speakers [with Cook's *D* > 4/*N*(speakers)], who produce only 2% of the total data in this age group. Detailed analysis of the data by these speakers shows that they are heavily dominated by single conversational particles (equivalent to English “oh,” “yes,” “no,” “huh?” etc.) that are coded as Nepali because they are identical with, but not necessarily borrowed from, Nepali (see the Supplementary Material for the relevant data). These particles are twice as frequent in the data of the 6 speakers than in the rest of the data (29.6% vs. 14.8% of one-word utterances). After removing the data of these speakers, no evidence is left for a significant increase of Nepali one-word utterances above age 25 (χ^2^ = 0.28, *P* = 0.596).

**Figure 3:**
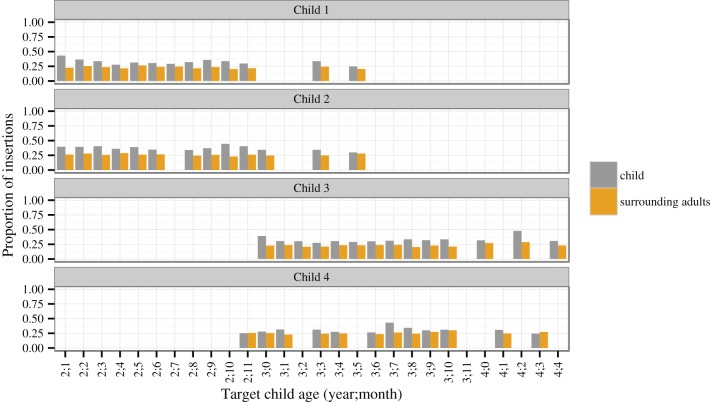
**Mean proportions of Nepali insertions per utterance over 1-month intervals: target children in the language acquisition corpus compared to the surrounding adults within the same recording session**. (Sample sizes in *N* utterances: child 1: 15,631, adults 1: 21,206; child 2: 17,149, adults 2: 24,147; child 3: 17,651, adults 3: 16,909, child 4: 13,163, adult 4: 21,194).

The increased proportion of Nepali insertions in children's longer utterances can be confirmed by zooming in into the language acquisition corpus, contrasting the productions of target children between age 2 and 4 with the adults surrounding them in each session (Figure 3). The difference between target children and adults does not change much over these 2 years (cf. a model with age and utterance length vs. with utterance length only: χ^2^ = 1.07, *P* = 0.3). But children show significantly higher proportions throughout for utterances with two (χ^2^ = 37.89, *P* < 0.001) or more words (χ^2^ = 27.03, *P* < 0.001); for one-word utterances, no difference is detectable (χ^2^ = 0.60, *P* = 0.44).

### 4.2. Single-word vs. multi-word insertions

**Figure 4:**
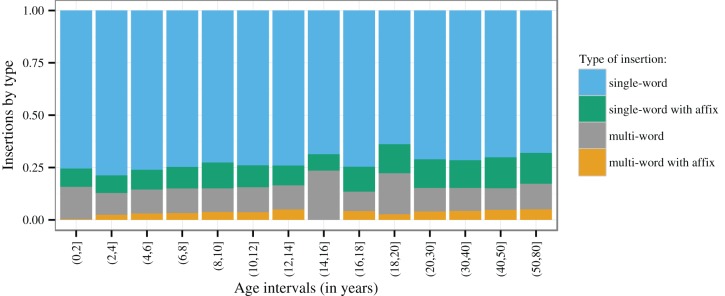
**Proportion of types of Nepali insertions: single-word insertions vs. multi-word sequence insertions, with or without Chintang morphology attached (Sample sizes in Table 1)**.

Figure 4 shows that single-word insertions outnumber multi-word sequence insertions by a mean factor of about 5.31 (*Standard deviation* = 0.96), averaging across ages. The figure also indicates which insertions show Chintang affixation (at least one affix in the string), i.e., a morphological signal of syntactic integration. While always in a minority, insertions with Chintang affixation make up a substantial proportion of all insertions, in both single-word and multi-word insertions: the proportions of insertions with affixation per session and speaker average at 14% for single-word insertions (mean *N* per session and speaker = 33.61) and at 25% for multi-word insertions across all ages (mean *N* = 7.42). Grand totals in the entire corpus are shown in Table 1.

**Table 1:** **Grand totals of Nepali insertion types with and without Chintang affixation (utterances with at least two words for single-word insertions and with at least three words with multi-word insertions)**.

	**Single word**	**Multiple words**
Without Chintang affix	69,712 (86%)	10,582 (75%)
With Chintang affix	11,180 (14%)	3,521 (25%)

In reality, the proportions of syntactically integrated insertions might be even higher because (as noted before) our measurement only captures insertions with morphological consequences. But not all integrations have such consequences since for example the nominative case has no visible morphological exponence whatsoever. Also, it is possible that the higher proportion of Chintang affixation in multi-word compared to single-word insertions is a stochastic side-effect of the fact that in multi-word insertions there are more occasions for affixation than in single-word insertions.

**Figure 5:**
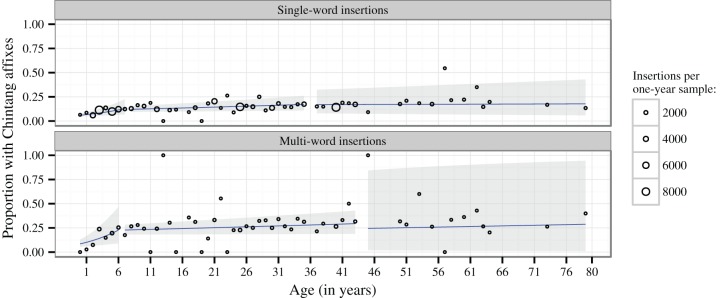
**Proportion of Nepali insertions with vs. without Chintang affixation over 1-year age intervals**. The size of dots is proportional to the number of insertions within these intervals. The regression lines (blue) and 95% Wald confidence intervals (gray) represent the fixed effects estimates from generalized linear mixed models applied to individual insertions and with recording session and speaker as random factors, assuming a binomial distribution. Breakpoints are those that minimize the deviance of the models.

Figure 5 plots the proportion of insertions with vs. without affixation over 1-year intervals, together with regression lines and 95% confidence intervals estimated by GLMMs modeling the presence vs. absence of Chintang affixes on each insertion as a logistic response to age.

A breakpoint analysis modeling of these GLMMS reveals a first significant breakpoint at age 7;2 for single-word insertions (χ^2^ = 23.991, *P* < 0.001) and at age 6;3 for multi-word insertions (χ^2^ = 21.14, *P* < 0.001). Below this age, there is a significant increase in the probability of affixation for both single-word insertions (χ^2^ = 15.36, *P* < 0.001) and multi-word insertions (χ^2^ = 12.45, *P* < 0.001). Above these breakpoints, affixation continues to increase significantly, albeit at a slower rate (χ^2^ = 11.57, *P* < 0.001 for single-word and χ^2^ = 4.20, *P* = 0.040 for multi-word insertions).

To assess the shape of this latter development we submitted the data for speakers older than 7;2 and 6;3 to a further breakpoint analysis. This revealed a significant breakpoint at age 35 for single-word insertions (χ^2^ = 4.80, *P* = 0.029) with a significant increase below this age (χ^2^ = 9.97, *P* = 0.002) and no further development above this age (χ^2^ = 0.08, *P* = 0.782). The best-fitting breakpoint for multi-word insertions was estimated at age 43, but this breakpoint does not significantly improve the fit of the GLMM (χ^2^ = 1.36, *P* = 0.243). Nevertheless, we found age to have a significant effect on affixation below this age (χ^2^ = 5.21, *P* = 0.022), but not above (χ^2^ = 0.21, *P* = 0.644).

**Figure 6:**
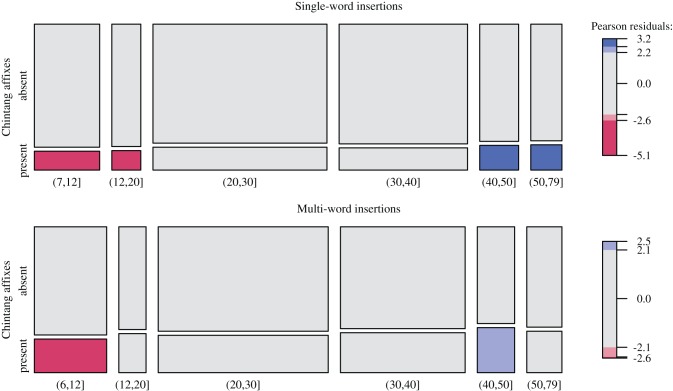
**Relative counts of insertions with vs. without Chintang affixation over age groups above 7 (single-word insertions) and 6 (multi-word insertions) years of age**. Tile size is proportional to counts and shading indicates the level at which Pearson residuals show a significant deviation from the null hypothesis of independence between age and counts, with light colors for α= 0.05 and darker colors for α= 0.01 [following the technique of Zeileis et al. (2007)]. Positive residuals appear in blue, negative residuals in red; gray signals statistical non-significance.

The change in affixation probabilities around age 35–43 is confirmed by an analysis of summary counts across age intervals. These are shown in the mosaic plots in Figure 6, where Pearson residuals are shaded according to the level at which they show a significant deviation from a null model of no interaction between age and counts [following the method suggested by Zeileis et al. (2007)]. Again, speakers older than 40 show significantly increased counts of insertions with affixation while school children and adolescents show significantly depressed counts.

## 5. Discussion

Our analysis of code-switching across generations reveals that Chintang is in a relatively stable shape and code-switching has relatively low over-all proportions, consistent with earlier findings which focused on a smaller sample and on the insertion of all-Nepali utterances only (Stoll et al., 2012). This finding suggests that Chintang is far away from being a mixed language like Michif (Bakker, 1997) or Media Lengua (Muysken, 1996), where utterances tend to be mixed to a much stronger extent.

The extent of code-switching is different for children below age 6–7. These children show significantly more Nepali insertions in two-word or longer utterances than older speakers and they also show a steady decrease until they reach the same levels as older speakers (Figure 2). This time frame and developmental signature is typical of an acquisition process. The pattern is unlikely to reflect a diachronic change since there is no robust evidence for a change across speaker generations after the acquisition process is completed. We do find some evidence of a cross-generational trend among one-word insertions, but as noted in the Results section, this signal is likely to be spurious. In general, one-word insertions do not appear to change during language acquisition or across generations. This is consistent with our proposal that the pattern below age 6–7 is developmental because single words are much easier to learn than longer constructions. Therefore, children reach adult performance in these utterances very early.

However, in the absence of long-term longitudinal data, we cannot of course completely rule out the possibility that the pattern in Figure 2 is caused by a diachronic change: it is possible in principle that today's adult generation started out with lower degrees of code-switching and that the present generation of preschoolers will keep their higher rate of insertions when they grow up. However, this kind of language change would have to be induced by preschoolers – a scenario that is unlikely unless the change is triggered by highly specific modifications in child-directed speech, such as what is reported for Gurindji and Warlpiri language change (McConvell and Meakins, 2005; O'Shannessy, 2005, 2012). We have no evidence for comparable modifications of child-directed speech in Chintang.

The developmental pattern in our data might reflect similar processes as have been found in studies of code-switching of children growing up in individual bilingual situations. For such situations, it has been proposed that a gradual decrease of code-switching reflects the fact that children slowly expand their first-language vocabularies and rely less on on-the-spot borrowings from whatever is offered in their input (Vihman, 1985). Our findings diverge from those reported for other situations of community-wide bilingualism such as Inuktitut/English (Allen et al., 2002) and French/English bilingualism (Paradis et al., 2000). However, an interpretation of this divergence is difficult because of substantial differences in corpus size, sampling regimes and various controls, such as individual ages of children and adults.

Another result of our study is that Chintang shows a substantial proportion of syntactically integrated insertions, as reflected by Chintang affixes on Nepali insertions (Figure 4). This characterizes not only single-word but also multi-word insertions where on average about 25% insertions per session show Chintang affixation (Table 1). These proportions develop gradually during acquisition, up to about age 6–7 (see the left-most regression lines in Figure 5). We take this gradual development to reflect the fact that Chintang affixation on Nepali insertions requires substantial command of Chintang morpho-syntax, a command that children are unlikely to reach to the same extent as adults before age 6 or 7. However, adult patterns are not homogenous: the data suggest a steady increase of integration probabilities until they reach their maximum after about age 35–43.

The adult patterns are surprising in light of received theory. It has often been argued in the literature that multi-word insertions differ strongly from single-word insertions with regard to syntactic integration (Poplack et al., 1988). These two types of switching are considered to rely on different cognitive mechanisms. Single-word insertions are considered instances of borrowing and are as such expected to trigger immediate integration into the native grammar. Multi-word insertions, by contrast, are considered to reflect full-fledged switching in language and grammar, leaving little room for integration of one language into the other (Poplack and Meechan, 1998a).

Yet in our data, multi-word insertions show as much integration as single-word insertions. While in the absence of full syntactic and semantic parsing of the corpus, we cannot exclude the possibility that some multi-word insertions are in fact idiomatic and lexicalized, compound-like sequences, a qualitative survey suggests that this type of multi-word insertions is far from being the dominant pattern. Also, as noted before, our measure of integration (affixation), inevitably underestimates the true amount of syntactically integrated insertions. Thus, even if one were able to weed out lexicalized multi-word insertions, there are likely to remain substantial numbers of syntactically integrated insertions in our dataset. This suggests that the length of insertions (single vs. multi-word) may not be a good criterion for distinguishing between proper insertion of one language into the other vs. code-switching between separate languages.

One possible explanation for the generally high degree of integration in our data might come from the kind of morphology that is involved. Nominal affixes are only loosely attached to their hosts in Chintang: case, number and possessive marking are affixes on syntactic phrases, not on lexical stems (and are thus very different from classical Indo-European-style word inflection). This can be seen for example in the fact that cases are only ever attached to the right edge of phrases and never percolate to dependents, e.g., to adjectives. An illustration of this can be seen in (4), where case markers attach to the entire inserted phrase (with a locative in [*euta sahayok*]*-be* “in one kind of help” and with an ergative in [*junai sastha*]*-ŋa = yaŋ* “whichever organization”). Exactly the same pattern holds for native phrases. Another effect of the fact that affixes are attached to phrases rather than to stems is the behavior of possessive prefixes which can appear on either the head noun or a dependent in a phrase, e.g., “my red house” can be expressed either as [*a-halochopma khim*] (“my-red house”) or as [*halochopma a-khim*] (“red my-house”). Most other grammatical markers on nouns are loosely bound clitics. Verbal morphology is characterized by phonological disintegration: while grammatically tightly bound, inflected forms typically consist of several distinct phonological words, with effects on morphophonology, endoclitic hosting and flexible positioning of prefixes (Bickel et al., 2007). Thus, overall Chintang morphology is relatively loose and flexible already in the native grammar. This might facilitate integration of Nepali insertions across the board.

Another factor that might explain the unusually high degree of syntactic integration is the fact that, despite all their typological differences, Chintang and Nepali show very similar word order and syntactic phrase structure rules. This makes mixing very easy, as it requires no major re-ordering during processing. In addition, the accidental similarity in form and distribution of a very frequent Chintang morpheme, the locative nominalizer *-ko*, with the Nepali participle and genitive marker *-ko* might help to blend structures. Both serve to mark, for instance, dependent nouns in expressions like Chintang *a-phuwa-ko khim* and Nepali *mero dai-ko ghar* “my elder brother's house”^5^. Such parallelisms could support the emergence of a single, convergent syntax at least in some areas of bilinguals' competence, similar to what has been proposed in the classic study by Gumperz and Wilson (1971) on convergence between Indo-Aryan and a Dravidian language. What may have further facilitated such a convergence in Chintang is that there is virtually no trace of a puristic language ideology that would favor a version of Chintang that is free from Nepali as much as possible. This is consistent with our ethnographic observation that the choice of languages does not seem be as critical a carrier of social identity as is often found in modern Western societies. In a neighboring and closely related community, the language that people speak does not even have a distinctive endonym and is labeled by speakers in the same way as the language of another, politically more important language (Bickel, 2003).

However, while these observations may explain the overall amount of integration we find compared to other languages, none of them accounts for the fact that there is a significant change in the extent of integration across generations. This difference is not accounted for by morphology or syntax because there is no evidence for a concomitant difference in morphology or syntax between these age groups. Children reach adult levels of proficiency in morphology at around age 4 (Stoll et al., 2012) and the developmental pattern of syntactic integration suggests that they have acquired a substantial part of adult morphosyntax by age 6 or 7. Also, we see no pattern of trans-generational change in word order rules or phrase structure patterns. Further, from a comparative perspective, Chintang morphology and syntax is likely to be old and consistent for all generations of the current population, indeed probably also many generations before them. In these regards, the Chintang situation is very different from the situation in Warlpiri or Gurindji (McConvell and Meakins, 2005; O'Shannessy, 2005, 2012). Finally, language ideology does not appear to have fundamentally changed over the past decades, although changes are more likely at present and in the future, as a result of large-scale political changes in Nepal.

These considerations suggest an alternative explanation of the change across Chintang generations: the difference between generations is not a change in Chintang, but a change in the nature of bilingualism. For speakers of older generations, the native Chintang grammar is fully dominant and deeply entrenched in the speaker's language competence. Conversely, the inserted language, here Nepali, is less entrenched and secondary. As a result, speakers can easily treat Nepali items as if they were native and coerce them into their native grammar. For speakers of the generations characterized by current youth, the situation is different. As noted in Section 2, Nepali became more prominent in recent years and this makes it likely that for current adolescents, Nepali has started to play a role that is almost as important as that of Chintang. In a more balanced system of this kind, one would expect the two grammars to be more equally entrenched representationally. This would result in a better entrenchment of both languages, with the result that they no longer interweave as easily as is possible in strongly asymmetric bilingualism.

Support for this theory comes from the fact that it is precisely the generation of today's speakers above age 35–43 that had considerably less exposure to Nepali in their childhood due to a lack of schooling. Schooling in Nepal is exclusively in Nepali and the use of Nepali extends to the surrounding of the schools. According to the Population Census of Nepal 1971 (Central Bureau of Statistics, 1977), schooling increased by nearly 60% during the preceding decade. This fact coincides with our breakpoint of generations for stronger integration of Nepali into Chintang: the generation older than 35–43 years had considerably less exposure to Nepali since schooling was limited. Day-to-day use of Nepali was mostly restricted to short interactions with Nepali native speakers.

To test this theory further, one would now need to systematically assess the development of Nepali competence and usage patterns among Chintang speakers of different generations. Further evidence could come from processing research on speakers of different generations. Neuro-imaging studies on language production (Perani et al., 2003) show that brain activation patterns vary among highly proficient bilinguals, critically depending (among other factors) on the age of acquisition and the amount of exposure of the second language. From our findings, we would expect that older speakers show systematically different activation patterns than speakers of younger generations, reflecting the different degrees of entrenchment of Nepali in the two groups. This, however, remains to be tested in future research.

## 6. Conclusion

This study confirms results from earlier research suggesting that young bilingual children show more code-switching than adults. This is likely to be caused by the fact that lexical competence in the native language is not yet fully developed and so children resort to whatever lexical choices are offered in the input, regardless of the language that the choices come from.

Our study also revealed an unexpected finding: even though Chintang speakers show a relatively low overall proportions of code-switching and the language is far away from being a mixed language, a substantial number of multi-word Nepali insertions are fully integrated into the native syntax, often triggering morphological reflexes of this integration. The extent of such integration is significantly higher for speakers of older generations. A possible explanation of this, which needs to be tested by further research, is that for these generations, native Chintang syntax heavily dominates language use, and Nepali plays only a weak role as a provider of lexical items. Younger speakers show less syntactic integration, which suggests that they have developed a more balanced bilingual competence, resulting in a stronger segregation of the two systems.

What has also become apparent from this study is that patterns of code-switching over generations are best detected in large-scale quantitative analyses of naturalistic corpora, as also suggested by Poplack (in press). Given the overall low proportion of code-switching in Chintang, a qualitative or small-corpus study might not have been able to detect the patterns of syntactic integration that we found and their change over generations.

## Author contributions

Study design and theory: Sabine Stoll, Balthasar Bickel, Robert Schikowski; Language data extraction and analysis: Robert Schikowski, Balthasar Bickel; Statistical analysis: Balthasar Bickel, Taras Zakharko, Sabine Stoll, Steven Moran; Corpus development: Sabine Stoll, Robert Schikowski, Taras Zakharko, Balthasar Bickel, Write-Up: Sabine Stoll, Balthasar Bickel.

## Funding

This research was supported by Volkswagen Foundation Grant Nos. II/79092 (2004-2009, PI Bickel) and II/81730 (2007-2012, PI Stoll) and by funding from the Max Planck Institute for Evolutionary Anthropology, Leipzig, Germany and the Comparative Linguistics Department, University of Zurich.

### Conflict of interest statement

The authors declare that the research was conducted in the absence of any commercial or financial relationships that could be construed as a potential conflict of interest.

## Footnotes

^1^Interlinear morpheme glossing follows the Leipzig Glossing Rules (http://www.eva.mpg.de/lingua/resources/glossing-rules.php). Note that elements in square brackets on the glossing line represent the meanings of zero morphemes. Abbreviations are as follows: A “most agentive argument,” ASS “assertive,” CLF “numeral classifier,” CONTR.TOP “contrastive topic,” ERG “ergative,” FOC “focus,” i “inclusive,” IND “indicative,” INF “infinitive,” IPFV “imperfective,” LOC “locative,” MED “medial demonstrative,” METHOD “method case,” NMLZ “nominalizer,” NPST “non-past,” ns “non-singular,” NTVZ “(generic) nativizer,” O “object,” OBL “oblique case,” OPT “optative,” p “plural,” PRF “perfect,” PROX “proximal demonstrative,” PST “past,” PTCP “participle,” QTAG “question tag,” RETRV “retrieval instruction,” s “singular,” S “sole argument of intransitive verb,” V.NTVZ “verbal nativizer.” Hyphens (“−”) represent affix boundaries (including phrasal affixes); equal signs (“=”) represent syntactically independent but phonologically bound words and particles (“clitics”).

^2^We write Nepali using standard orthography in Indological transliteration. Short 〈*a*〉 is typically realized as [ʌ]; long 〈*ā*〉 as [a].

^3^The sub-corpus used in our analysis is available as Supplementary Material. Because of the sensitivity of the actual content (gossip, politics etc), we removed all content and speaker names and pared down the corpus to the actual information processed in the analysis (language source per morpheme, speaker age etc.). Data collection and curation followed the ethics guidelines of the linguistics departments at the Max Planck Institute for Evolutionary Anthropology and at the University of Leipzig, the institutions where the PIs of the project (Stoll and Bickel) worked at the time (http://www.eva.mpg.de/linguistics/resources/ethics-guidelines.html; http://www.uni-leipzig.de/~ff/ethicguidelines.html).

^4^Unless indicated otherwise, there is one degree of freedom in the likelihood ratio test results that we report.

^5^Chintang *-ko* is historically unrelated to Nepali *-ko*. The Chintang marker is composed of a locative in *-(i)* and a nominalizer in *-go* ~ *-ko*, a structure that shows up explicitly in alternative forms like *-(i)-ko*.
